# Research on exosomes in cancer multidrug resistance and clinical translation

**DOI:** 10.20517/evcna.2025.191

**Published:** 2026-05-19

**Authors:** Wenxin Zhang, Mengyuan He, Xiaoli Cheng, Qingqing Chai, Aixue Li, Fengna Li, Yongwei Gu, Kang Fang, Rong Rong, Jiyong Liu

**Affiliations:** ^1^College of Pharmacy, Shandong University of Traditional Chinese Medicine, Jinan 250355, Shandong, China.; ^2^State Key Laboratory of Neurology and Oncology Drug Development, Nanjing 210000, Jiangsu, China.; ^3^Department of Pharmacy, Fudan University Shanghai Cancer Center, Shanghai 200032, China.; ^4^School of Pharmacy, Faculty of Medicine Macau University of Science and Technology Macau, Macau 999078, China.; ^5^Department of Pharmacy, Huashan Hospital, Fudan University, Shanghai 200040, China.; ^#^Authors contributed equally.

**Keywords:** Exosomes, multidrug resistance, tumor microenvironment, therapy resistance

## Abstract

Multidrug resistance (MDR) is a major clinical challenge that limits the efficacy of multiple cancer treatment modalities, including chemotherapy, targeted therapy, immunotherapy, monoclonal antibody therapy, and antibody-drug conjugates. In recent years, exosomes (Exos), nanoscale vesicles involved in intercellular communication, have attracted increasing attention for their roles in the formation and spread of MDR. A growing body of evidence suggests that Exos mediate the transfer of resistance-related molecular signals among drug-resistant cancer cells, drug-sensitive cancer cells, and stromal cells, such as cancer-associated fibroblasts and tumor-associated macrophages, through the selective packaging of noncoding RNAs, functional proteins, and metabolic regulators. These molecular signals may induce the reprogramming of signaling pathways, metabolism, and epigenetic states in recipient cells, thereby promoting the acquisition of cancer stem cell-like properties and a drug-resistant phenotype. In turn, these changes may contribute to the establishment of a drug resistance-supporting tumor microenvironment. This review systematically summarizes the molecular mechanisms by which Exos contribute to multidrug resistance, with a particular focus on their roles in cargo sorting, microenvironmental crosstalk, and the functional reprogramming of recipient cells. It also discusses their potential for clinical translation in resistance monitoring and reversal therapy. In addition, this review further discusses the key challenges currently facing the field and provides perspectives on future research directions.

## INTRODUCTION

Cancer remains one of the leading causes of death and a major threat to human health^[1]^. Existing therapeutic strategies have considerable limitations^[2,3]^. Although surgery, chemotherapy, and radiotherapy are often cause significant adverse effects, and some patients still experience limited long-term benefit or disease recurrence^[2-7]^. Molecularly targeted therapy can reduce the adverse effects of chemotherapy and radiotherapy, however, sustained therapeutic pressure may drive tumor cells to develop resistance through secondary genetic mutations or through compensatory activation of signaling pathways^[8-10]^. Immunotherapy partially overcomes these limitations by activating antitumor immune cells. However, immunoregulatory function is often impaired in advanced cancers^[11-14]^. Conventional strategies to overcome drug resistance, such as combination therapy and dose escalation, are often limited by tumor heterogeneity, adaptive signaling reprogramming, and protective effects mediated by the tumor microenvironment (TME)^[15-18]^. Moreover, resistance is not limited to conventional chemotherapeutic agents such as anthracyclines, platinum compounds, taxanes, and antimetabolites. Molecularly targeted agents such as tyrosine kinase inhibitors, as well as immune checkpoint inhibitors, therapeutic monoclonal antibodies, and antibody-drug conjugates (ADCs), also face the critical challenge of primary or acquired resistance in clinical practice^[19-23]^. Drug resistance not only compromises the initial therapeutic response and leads to treatment failure, but is also closely associated with disease progression, recurrence, metastasis, and increased risk of death. Consequently, it represents a major clinical bottleneck that limits long-term survival benefits in cancer^[2,6,17,24]^. Therefore, there is a need to develop novel strategies that overcome drug resistance while improving therapeutic specificity and efficacy, and to re-examine resistance development from the perspective of tumor microenvironmental communication.

Accumulating evidence suggests that tumor multidrug resistance (MDR) is not merely an intrinsic event of individual tumor cells but it can be viewed as a “networked” phenotype shaped by the coordinated interactions and co-evolution of multiple cell types within the TME. This process depends on close communication among tumor cells, stromal cells, and immune cells^[25]^. In this context, exosomes (Exos) have attracted widespread attention because of their intrinsic delivery capacity and roles in intercellular communication. These membrane vesicles of approximately 30-150 nm in diameter are released upon fusion of multivesicular bodies with the plasma membrane^[26-28]^. As important mediators of intercellular communication within the TME, Exos carry specific biological information from their cells of origin. Their cargo includes proteins, lipids, metabolites, and various nucleic acid molecules, such as microRNAs (miRNAs), long non-coding RNAs (lncRNAs), and circular RNAs (circRNAs). Consequently, Exos have attracted considerable interest in studies of tumor initiation and progression, drug resistance dissemination, and clinical translation^[29-33]^. Given their unique roles in intercellular communication and the delivery of resistance-related biological information, Exos are increasingly recognized as an important lens through which to understand the formation and dissemination of tumor multidrug resistance.

This review summarizes the multi-layered mechanisms through which Exos contribute to multidrug resistance, with a particular focus on their roles in selective cargo sorting and loading, TME intercellular crosstalk, and the functional reprogramming of recipient cells. These processes are closely associated with the formation and maintenance of a drug-resistant phenotype. Some experimental studies further suggest that exos may promote the acquisition of cancer stem cell-like (CSC-like) properties and a drug-resistant phenotype in drug-sensitive cells. In addition, this review discusses the potential value of Exos in dynamic resistance monitoring and precision intervention from both mechanistic and application-oriented perspectives. It aims to provide insights for further development and clinical translation of related diagnostic technologies and mechanism-driven therapeutic strategies.

## THE MECHANISTIC ROLE OF EXOS IN MDR

Current evidence suggests that the involvement of Exos in MDR may constitute a continuous process linking cargo loading, intercellular transfer, and recipient cell reprogramming. First, resistance-related noncoding RNAs (ncRNAs), proteins, and metabolic regulatory molecules are selectively sorted and packaged into Exos^[34-37]^. Subsequently, Exos mediate information exchange among tumor cells, stromal cells, and immune cells within the TME. Ultimately, these cargoes are delivered to recipient cells, where they influence signaling pathways, metabolic states, and phenotypic characteristics, thereby promoting the formation and maintenance of the drug-resistant phenotype^[34,38]^. Therefore, the selective sorting and loading of cargoes are considered important early events in exosome involvement in MDR.

### Exosomal cargo and MDR-related information transfer

The intercellular transmission of the multidrug-resistant phenotype is closely associated with the ability of Exos to carry and transport specific bioactive cargoes^[35]^. Encapsulated within a lipid bilayer, these cargoes remain relatively stable in the complex TME, enabling delivery to both local and distant cells^[39-41]^. Current evidence indicates that nucleic acids, proteins, and metabolism-related molecules are important functional cargoes involved in exosome-mediated drug resistance. Different cargo types may drive the formation and maintenance of the drug-resistant phenotype through distinct molecular mechanisms^[42-45]^.

#### Exosome-delivered functional cargoes and MDR phenotype formation

Within the TME, stromal cells or drug-resistant cells may deliver bioactive molecular cargo to drug-sensitive cells through Exos, thereby promoting the dissemination of the drug-resistant phenotype. These functional cargoes include nucleic acids, proteins, and metabolism-related molecules. Through distinct mechanisms, they may influence the functional state of recipient cells and promote the formation and maintenance of the drug-resistant phenotype^[46-49]^.

Nucleic acid molecules are important mediators of gene regulation. ncRNAs are the most extensively studied effector molecules and include miRNAs, lncRNAs, and structurally stable circRNAs. For example, in pancreatic cancer (PC), Exos can induce gemcitabine resistance by delivering miR-155, which downregulates deoxycytidine kinase (DCK)^[46]^. In addition, exosomal delivery of lncRNA PSMA3-AS1 from bone marrow mesenchymal stem cells can promote resistance to proteasome inhibitors in multiple myeloma by upregulating PSMA3-related pathways^[47]^. Moreover, in hepatocellular carcinoma, Exos can mediate sorafenib resistance by delivering circRNA-SORE, which stabilizes Y-box binding protein 1 (YBX1)^[48]^. Once delivered via Exos, these nucleic acid molecules regulate gene expression and related signaling pathways in recipient cells, thereby promoting the formation of the drug-resistant phenotype. Representative studies on exosomal ncRNAs mediating drug resistance are summarized in Table 1.

**Table 1:** Exosomal ncRNAs mediating drug resistance

**Tumor type**	**Source of Exos**	**Key ncRNA cargo**	**Recipient cells**	**Major target/pathway**	**Drug resistance-related effects**	**References**
PC	Exos derived from drug-resistant PC cells	miR-155	Drug-sensitive PC cells	miR-155/DCK axis	Promotes gemcitabine resistance	[46]
MM	Exos derived from BMSCs	PSMA3-AS1	MM cells	PSMA3-AS1/PSMA3/proteasome pathway	Promotes resistance to proteasome inhibitors	[47]
HCC	Exos derived from sorafenib-resistant HCC cells	circRNA-SORE	Sorafenib-sensitive HCC cells	Stabilization of YBX1 by blocking PRP19-mediated degradation	Spreads and sustains sorafenib resistance	[48]
CRC	Exos derived from CAFs	miR-92a-3p	CRC cells	miR-92a-3p/FBXW7, MOAP1/Wnt/β-catenin/mitochondrial apoptosis pathway	Promotes resistance to 5-FU and oxaliplatin	[50]
CRC	Exos derived from CAFs	lncRNA H19	CRC cells	H19/miR-141/β-catenin axis	Promotes stemness and chemoresistance	[51]
GC	Exos derived from M2 TAMs	lncRNA MALAT1	GC cells	MALAT1/β-catenin and miR-217-5p/HIF-1α axis	Promotes chemoresistance	[52]
Cervical cancer	Exos derived from TAMs/M2-like macrophages	miR-660-5p	Cervical cancer cells	miR-660-5p/ALOX15 axis	Enhances resistance to ferroptosis-inducing therapies	[53]
NSCLC	Exos derived from NSCLC cells	circUSP7	CD8+ T cells	circUSP7/miR-934/SHP2 axis	Induces CD8+ T-cell exhaustion and promotes resistance to anti-PD-1 therapy	[54]
AML	Exos derived from AML cells	circ_0006896	CD8+ T cells	circ_0006896/HDAC1/LEF1 axis	Induces CD8+ T-cell dysfunction, reduces chemosensitivity, and promotes immune evasion	[55]
CRC	Exos derived from oxaliplatin-resistant CRC cells	miR-208b	CD4+ T cells/Tregs	miR-208b/PDCD4 axis	Promotes Treg expansion and immune evasion, thereby enhancing oxaliplatin resistance	[56]
CRC	Exos derived from oxaliplatin-resistant CRC cells	circATG4B	Oxaliplatin-sensitive CRC cells	circATG4B-222aa/TMED10/ATG4B/autophagy pathway	Promotes oxaliplatin resistance	[57]
MM	Exos derived from BMSCs	miR-155	MM cells	Hedgehog signaling/MRP1/ABCG2/P-gp	Maintains stemness and promotes drug resistance	[58]
MM	Exos derived from myeloma-associated adipocytes	lncRNAs LOC606724 and SNHG1	MM cells	Inhibition of drug-induced apoptosis; cargo loading promoted by METTL7A-mediated m6A modification	Enhances tolerance to bortezomib and other agents	[59]
CRC	Exos derived from oxaliplatin-resistant CRC cells	circ_0001610	Oxaliplatin-sensitive CRC cells	circ_0001610/miR-30e-5p/PGC-1α/OXPHOS axis	Enhances stemness and promotes oxaliplatin resistance	[60]

ncRNA: Noncoding RNA; PC: pancreatic cancer; MM: multiple myeloma; HCC: hepatocellular carcinoma; CRC: colorectal cancer; GC: gastric cancer; NSCLC: non-small cell lung cancer; AML: acute myeloid leukemia; BMSC: bone marrow mesenchymal stem cell; CAF: cancer-associated fibroblast; TAM: tumor-associated macrophage; Treg: regulatory T cell; 5-FU: 5-fluorouracil; DCK: deoxycytidine kinase; YBX1: Y-box binding protein 1; PRP19: pre-mRNA processing factor 19; FBXW7: F-box and WD repeat domain-containing 7; MOAP1: modulator of apoptosis 1; HIF-1α: hypoxia-inducible factor 1α; ALOX15: arachidonate 15-lipoxygenase 15; SHP2: Src homology 2 domain-containing phosphatase 2; HDAC1: histone deacetylase 1; LEF1: lymphoid enhancer-binding factor 1; PDCD4: programmed cell death 4; TMED10: transmembrane emp24 domain-containing protein 10; ATG4B: autophagy-related 4B cysteine peptidase; MRP1: multidrug resistance-associated protein 1; ABCG2: ATP-binding cassette subfamily G member 2; P-gp: P-glycoprotein; METTL7A: methyltransferase-like 7A; m6A: N6-methyladenosine; PGC-1α: peroxisome proliferator-activated receptor gamma coactivator 1-alpha; OXPHOS: oxidative phosphorylation.

Protein cargoes enable more direct functional transfer compared to nucleic acids. Exos can directly transport drug efflux pumps, such as P-glycoprotein (P-gp), thereby directly enhancing the drug-resistant capacity of recipient cells^[61]^. In addition, Exos can deliver signaling pathway-related proteins that activate bypass survival signaling. For example, in non-small cell lung cancer (NSCLC), exosomal transfer of wild-type epidermal growth factor receptor (wtEGFR) to recipient cells can activate a bypass signaling pathway that is not inhibited by osimertinib^[62]^. Similarly, Exos enriched with Wnt family member 3A (WNT3A) can activate the Wnt/β-catenin pathway and induce a drug resistance-associated stem-like phenotype in recipient cells^[63,64]^.

Metabolism-related molecules represent another important class of exosomal cargo involved in functional reprogramming. By delivering metabolic enzymes, Exos can alter the metabolic state of recipient cells and thus serve as important carriers of metabolic reprogramming. For example, in NSCLC, Exos derived from drug-resistant cells can transfer the key glycolytic enzyme pyruvate kinase M2 (PKM2) to drug-sensitive cells, thereby promoting glycolytic reprogramming and enhancing cisplatin tolerance^[49,65]^. Representative studies on exosomal proteins and metabolic factors mediating drug resistance are summarized in Table 2.

**Table 2:** Exosomal proteins and metabolic factors mediating drug resistance

**Tumor type**	**Source of Exos**	**Key protein/metabolic factor**	**Recipient cells**	**Major target/pathway**	**Drug resistance-related effects**	**References**
NSCLC	Exos derived from hypoxia-induced cisplatin-resistant NSCLC cells	PKM2	Cisplatin-sensitive NSCLC cells	Promotes glycolytic reprogramming and inhibits apoptosis via the PKM2/BCL2 axis	Promotes the transfer of cisplatin resistance	[49]
Breast cancer	Exos derived from DOC-resistant breast cancer cells	P-gp	Drug-sensitive breast cancer cells	Exosome-mediated P-gp transfer and drug efflux	Spreads the drug-resistant phenotype and enhances chemoresistance	[61]
NSCLC	Exos derived from EGFR–non-mutant resistant NSCLC cells	wtEGFR	EGFR-mutant, osimertinib-sensitive NSCLC cells	Activates the PI3K/AKT and MAPK pathways	Promotes osimertinib resistance	[62]
NSCLC	Exos derived from NSCLC cells	WNT3A	NSCLC cells	WNT3A/Wnt/β-catenin pathway	Enhances stemness and drug resistance–related phenotypes	[63]
CRC	Exos derived from fibroblasts/CAFs	Wnt proteins	Differentiated CRC cells	Activates Wnt signaling and induces dedifferentiation into CSC-like cells	Promotes chemoresistance	[64]
CRC	Exos derived from CRC cells	ADAR1	TAMs	ADAR1/AZIN1-GLI1 RNA editing and the SPP1/NF-κB pathway	Promotes oxaliplatin resistance	[66]

DOC: Docetaxel; P-gp: P-glycoprotein; NSCLC: non-small cell lung cancer; PKM2: pyruvate kinase M2; BCL2: B-cell lymphoma 2; EGFR: epidermal growth factor receptor; wtEGFR: wild-type EGFR; PI3K: phosphoinositide 3-kinase; AKT: protein kinase B; MAPK: mitogen-activated protein kinase; WNT3A: Wnt family member 3A; CRC: colorectal cancer; CAF: cancer-associated fibroblast; CSC: cancer stem cell; ADAR1: adenosine deaminase acting on RNA 1; TAM: tumor-associated macrophage; AZIN1: antizyme inhibitor 1; GLI1: GLI family zinc finger 1; SPP1: secreted phosphoprotein 1; NF-κB: nuclear factor kappa B.

#### Exosome-delivered functional cargoes and drug resistance reversal

Exos may exert bidirectional regulatory effects, as they can serve not only as carriers of cargoes that promote drug resistance, but also those reverse drug resistance or enhance drug sensitivity^[67]^. Exos carrying chemosensitizing cargoes may originate from two major sources. First, they may be derived from non-tumor cells. For example, Exos derived from bone marrow mesenchymal stem cells (BMSCs) can deliver miR-193a to cisplatin-resistant NSCLC cells, where it suppresses leucine-rich repeat-containing 1 (LRRC1) expression by directly targeting it, thereby increasing cisplatin sensitivity and partially reversing the drug-resistant phenotype^[68]^. Second, Exos derived from drug-sensitive cells or those enriched in chemosensitizing molecules may also mediate the intercellular transfer of molecular signals associated with drug sensitivity. For example, in triple-negative breast cancer (TNBC), Exos enriched in circRNA-CREIT can enter drug-resistant cells, promote the ubiquitination and degradation of protein kinase R (PKR), inhibit stress granule assembly, and activate the RACK1/MTK1 apoptotic pathway, thereby partially reversing doxorubicin resistance^[69,70]^.

#### Mechanisms of selective cargo sorting in Exos

Cargo sorting and packaging into Exos is a complex process that involves not only active sorting via recognition of specific molecular signals, but also potential passive encapsulation during biogenesis. For many molecules with well-defined functions, entry into Exos may depend on multilayered active sorting mechanisms^[37,71]^. This process can influence the specificity and efficiency of information transfer. First, RNA-binding proteins (RBPs) play important roles in the selective sorting of specific nucleic acid cargoes through the recognition and capture of particular nucleic acid molecules. For example, Y-box binding protein 1 (YBX1) can bind specifically to miR-223 and promote its sorting into Exos^[72]^. Second, post-transcriptional RNA modifications serve as important recognition signals within regulatory networks. RNA modifications themselves, such as N6-methyladenosine (m6A), can participate in selective sorting into Exos through recognition by proteins such as heterogeneous nuclear ribonucleoprotein A2/B1 (HNRNPA2B1)^[73]^. In addition, post-translational modifications of RBPs themselves, such as SUMOylation, can further refine this process. For example, SUMOylation of hnRNPA2B1 can affect its recognition and binding to miRNA exosomal sorting motifs, thereby promoting the incorporation of specific miRNAs into Exos^[74]^. Furthermore, cellular states and microenvironmental signals can dynamically regulate cargo sorting. For example, hypoxia can promote the enrichment of molecules such as miR-135b in Exos, which in turn enhances angiogenesis and remodels the tumor microenvironment by targeting factors inhibiting HIF-1 (FIH-1). This finding suggests that microenvironmental signal-driven remodeling of exosomal cargo may indirectly contribute to the formation of a drug resistance-related microenvironment^[75]^. In addition, the ubiquitination and deubiquitination of protein cargoes constitute an important regulatory layer in their sorting. Existing evidence suggests that ubiquitin-related modifications are involved in the incorporation of certain proteins into Exos, whereas COP9 signalosome subunit 5 (CSN5) can regulate this process through its deubiquitinating activity. Loss of CSN5 function leads to increased enrichment of proteins such as heat shock protein 70 (HSP70) in Exos, suggesting that the ubiquitin-related modification status of protein cargoes influences their loading^[76]^. Finally, in addition to the classical endosomal sorting complex required for transport (ESCRT) pathway, alternative sorting routes based on lipid-protein interactions also exist. Studies have shown that the interaction between ALG-2-interacting protein X (ALIX) and lysobisphosphatidic acid (LBPA) mediates a noncanonical sorting mechanism in which ALIX directly recruits ESCRT-III through its BRO1 domain, bypassing the classical upstream ESCRT complexes, thereby promoting the sorting of tetraspanins such as CD9, CD63, and CD81 into Exos^[77]^. Overall, although exosome biogenesis is accompanied by a certain degree of stochasticity, current evidence generally suggests that the loading of functionally relevant cargoes is largely regulated by active sorting mechanisms^[37]^.

### Exosome-mediated cellular crosstalk in the TME

#### Crosstalk between tumor cells and cancer-associated fibroblasts mediated by tumor cell-derived Exos

The crosstalk between tumor cells and cancer-associated fibroblasts (CAFs) mediated by Exos is a bidirectional and dynamic process. On the one hand, tumor cells can deliver specific molecules, such as miR-21, via Exos to remodel stromal fibroblast function, driving their conversion toward a pro-tumorigenic CAF-like phenotype^[78]^. On the other hand, CAFs can deliver various bioactive molecules to tumor cells through Exos, thereby promoting tumor cell survival, stemness maintenance, and activation of drug resistance-associated pathways^[79]^. For example, in colorectal cancer, Exos derived from CAFs can deliver miR-92a-3p, which, by suppressing F-box and WD repeat domain-containing 7 (FBXW7) and modulator of apoptosis 1 (MOAP1), activates Wnt/β-catenin signaling and inhibits mitochondrial apoptosis, thereby promoting the acquisition of cancer stem cell-like features and enhancing tumor cell tolerance to 5-fluorouracil (5-FU) and oxaliplatin^[50]^. Similarly, CAFs can also transfer lncRNA H19 via Exos. Acting as a sponge for miR-141, H19 likewise activates the Wnt/β-catenin pathway, thereby enhancing tumor stemness and promoting chemoresistance^[51]^. In addition, metabolic support between CAFs and tumor cells represents another important dimension of their crosstalk. Studies have shown that Exos derived from CAFs can deliver metabolism-related cargoes that help tumor cells undergo metabolic reprogramming under stress conditions, thereby maintaining their survival advantage^[80]^.

#### Exosome-mediated bidirectional regulation between tumor cells and immune cells

Exos are considered important mediators linking resistance to chemotherapy, targeted therapy, and immunotherapy. By modulating immune cells within the TME, Exos help establish an immunosuppressive niche, thereby promoting the formation and maintenance of MDR^[25,81]^. In this process, tumor cell-derived Exos can reprogram immune cell function, while reprogrammed immune cells, particularly tumor-associated macrophages (TAMs), can in turn act on tumor cells via Exos, thereby forming a bidirectional regulatory network associated with drug resistance^[82]^.

First, in TAM-related pathways, studies have shown that tumor cells can promote the polarization of macrophages toward a pro-tumorigenic M2-like phenotype via Exos and induce their functional reprogramming, thereby driving the remodeling of a microenvironment that supports drug resistance. In CRC, tumor-associated macrophages with high adenosine deaminase acting on RNA 1 (ADAR1) expression can promote the secretion of secreted phosphoprotein 1 (SPP1) by catalyzing RNA editing of GLI family zinc finger 1 (GLI1). SPP1 subsequently acts on cancer cells to activate the NF-κB signaling pathway, ultimately enhancing their tolerance to oxaliplatin^[66]^. In gastric cancer (GC), M2-polarized TAMs can transfer lncRNA MALAT1 to cancer cells via Exos. On the one hand, MALAT1 directly binds to and stabilizes δ-catenin, thereby preventing β-transducin repeat-containing protein (β-TRCP)-mediated ubiquitin-dependent degradation and activating the β-catenin signaling pathway. On the other hand, acting as a competing endogenous RNA (ceRNA), MALAT1 sponges miR-217-5p, leading to upregulation of hypoxia-inducible factor 1-alpha (HIF-1α). Together, these two pathways synergistically promote aerobic glycolysis in cancer cells, ultimately enhancing their tolerance to oxaliplatin^[52]^. In addition, in cervical cancer, Exos derived from TAMs can deliver miR-660-5p to target and downregulate arachidonate 15-lipoxygenase (ALOX15), a key enzyme involved in ferroptosis, thereby inhibiting ferroptosis in cancer cells and enhancing their survival advantage^[53]^. Beyond TAMs, Exos can also reshape T-cell function at the level of adaptive immunity. Exos secreted by tumor cells can carry programmed death-ligand 1 (PD-L1) on their surface. Upon binding to programmed cell death protein 1 (PD-1) on T cells, exosomal PD-L1 transmits inhibitory signals that impair T-cell function and promote tumor immune evasion. In addition, exosomal PD-L1 is associated with the response to immune checkpoint blockade. Studies have shown that inhibiting exosomal PD-L1 can restore antitumor immunity in models resistant to anti-PD-L1 antibodies, suggesting that it may be involved in both primary and acquired therapeutic resistance^[83,84]^. Exos can also reshape T-cell function at the post-transcriptional level by delivering genetic materials such as ncRNAs. For example, circUSP7 delivered by Exos derived from NSCLC cells can upregulate Src homology 2 domain-containing phosphatase 2 (SHP2) by sponging miR-934, thereby suppressing CD8+ T-cell effector function and promoting resistance to anti-PD-1 therapy^[54]^. Another study suggests that Exos derived from acute myeloid leukemia (AML) cells can deliver circ_0006896, which interacts with histone deacetylase 1 (HDAC1) in T cells and suppresses lymphoid enhancer-binding factor 1 (LEF1) transcription, thereby weakening T cell-mediated antitumor immunity^[55]^. In addition, Exos can indirectly suppress T cell-mediated antitumor immunity by altering the composition of immune cells within the TME. For example, oxaliplatin-resistant CRC cells can deliver miR-208b via Exos to drive the expansion of regulatory T cells (Tregs), thereby establishing a local immunosuppressive microenvironment and promoting oxaliplatin resistance^[56]^. Overall, exosome-mediated bidirectional regulation between tumor cells and immune cells is not a unidirectional process of immunosuppression, but rather a dynamic interactive network. This process is considered to be closely associated with the formation and maintenance of a microenvironment that supports drug resistance and may support tumor cells in acquiring survival advantages and therapy-resistant phenotypes.

#### Exosome-mediated drug resistance transmission: homotypic and heterotypic spread

Homotypic dissemination is one of the major modes by which Exos mediate the horizontal spread of the drug-resistant phenotype within tumor cell populations. Drug-resistant cells can transfer molecules such as circRNA-SORE^[48]^ and circATG4B^[57]^ to drug-sensitive cells via Exos, thereby promoting the acquisition of a drug-resistant phenotype in recipient cells. In contrast to homotypic spread, heterotypic dissemination involves stromal cells within the TME that serve as important sources of exosome-mediated drug resistance. Among these, BMSCs represent the most common resistance-inducing sources, as they can deliver specific ncRNAs to myeloma cells via Exos, thereby influencing drug resistance-related pathways. For example, in multiple myeloma (MM), Exos derived from BMSCs can deliver miR-155 to myeloma cells. This miRNA upregulates Hedgehog signaling and stemness-related molecules. In addition, it induces the expression of multidrug resistance-associated proteins such as P-glycoprotein (P-gp), multidrug resistance-associated protein 1 (MRP1), and ATP-binding cassette subfamily G member 2 (ABCG2), thereby promoting the formation of the drug-resistant phenotype^[58]^.

### Exosome-mediated reprogramming of recipient cells

**Figure 1:**
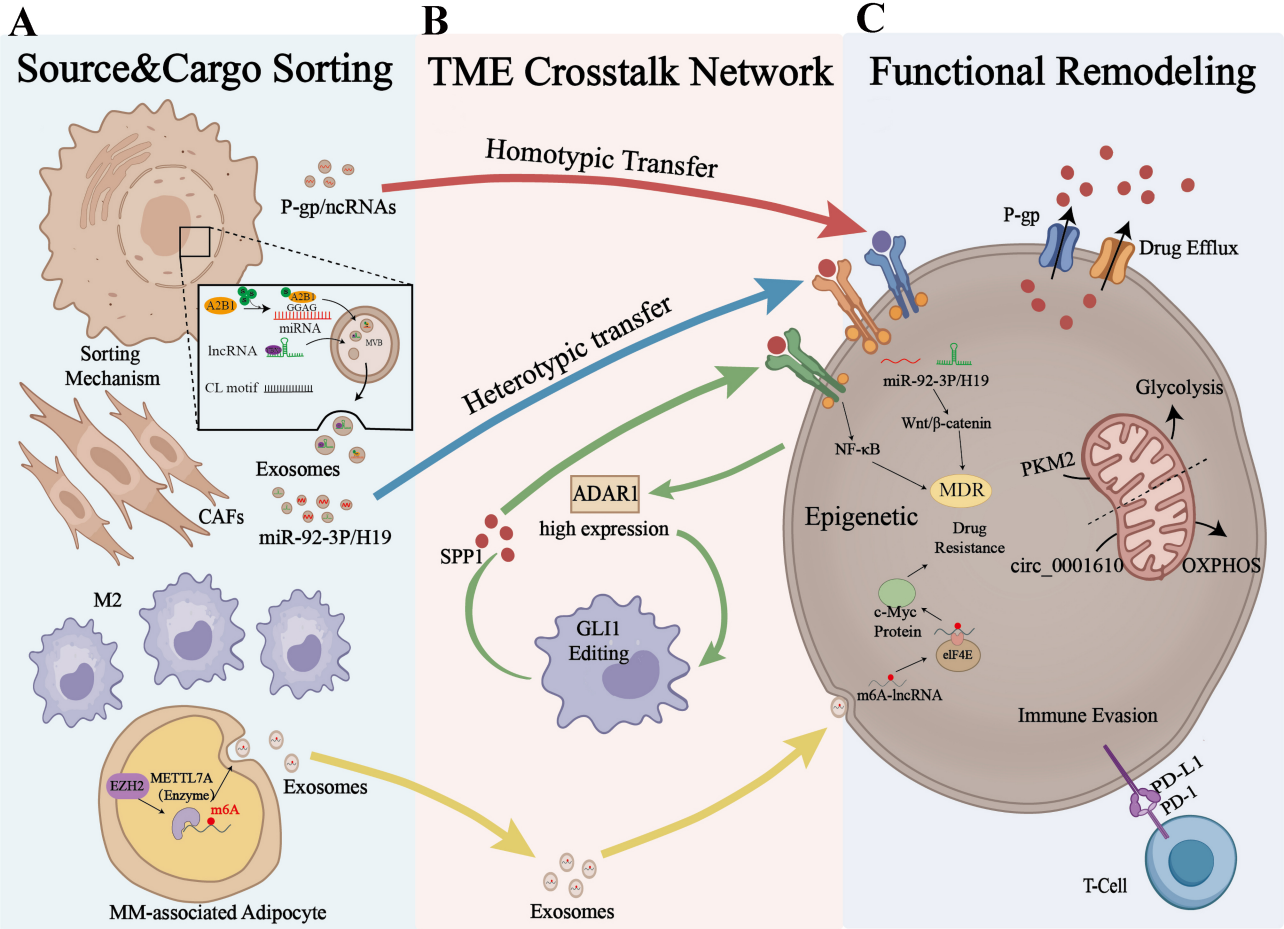
Schematic illustration of the major mechanisms by which Exos participate in tumor multidrug resistance. (A) Sorting and loading of exosomal cargo. Drug resistance-related cells can selectively enrich ncRNAs, functional proteins, and metabolism-related molecules and package them into Exos with the involvement of RNA-binding proteins (such as YBX1 and hnRNPA2B1) and RNA modifications; (B) Exos mediate the transmission of drug resistance-related information among drug-resistant cells, drug-sensitive cells, and tumor microenvironment-associated cells, and influence the functional states of CAFs, TAMs, and T cells, thereby forming a pro-resistance intercellular communication network; (C) Functional alterations in recipient cells. After uptake of Exos, recipient cells may undergo a variety of drug resistance-related molecular and phenotypic changes, including activation of classical signaling pathways, epitranscriptomic regulation, metabolic reprogramming, and inhibition of programmed cell death, which may further promote stemness maintenance and therapeutic resistance. This figure was created by the authors using Adobe Illustrator. Exos: Exosomes; ncRNAs: noncoding RNAs; YBX1: Y-box binding protein 1; hnRNPA2B1: heterogeneous nuclear ribonucleoprotein A2/B1; CAFs: cancer-associated fibroblasts; TAMs: tumor-associated macrophages; P-gp: P-glycoprotein; lncRNA: long non-coding RNA; CL: cellular-localization; EZH2: enhancer of zeste homolog 2; m6A: N6-methyladenosine; TME: tumor microenvironment; ADAR1: adenosine deaminase acting on RNA 1; SPP1: secreted phosphoprotein 1; GLI1: GLI family zinc finger 1; NF-κB: nuclear factor kappa B; MDR: multidrug resistance; PKM2: pyruvate kinase M2; OXPHOS: oxidative phosphorylation; PD-L1: programmed death-ligand 1; PD-1: programmed cell death protein 1.

One important mechanism by which Exos mediate MDR is their ability to influence the functional state of recipient cells through the delivery of bioactive molecules. Upon delivery to recipient cells via Exos, pro-resistance cargoes may induce reprogramming at the levels of signaling pathways, metabolism, and epigenetic regulation, thereby promoting the transition of recipient cells from a drug-sensitive to a drug-resistant state^[85-87]^ [Figure 1].

#### Exosome-mediated remodeling of key signaling pathways

Within the tumor microenvironment, Exos serve as important carriers of intercellular communication. Through the selective delivery of proteins, ncRNAs, and other molecular cargoes, they can influence key signaling pathways in recipient cells and thereby contribute to the regulation of tumor progression and therapeutic responses. For example, Exos enriched in miR-21 and secreted by bladder cancer cells can be taken up by macrophages. By suppressing phosphatase and tensin homolog (PTEN) and activating PI3K/Akt signaling and its downstream signal transducer and activator of transcription 3 (STAT3) pathway, they induce polarization toward an M2/TAM phenotype, thereby promoting tumor invasion and metastasis^[88]^. Meanwhile, Exos derived from M2-TAMs can deliver lncRNA MALAT1 to tumor cells. MALAT1 suppresses β-transducin repeat-containing protein (β-TRCP)-mediated ubiquitin-dependent degradation of δ-catenin and also acts as a sponge for miR-217-5p to upregulate hypoxia-inducible factor 1-alpha (HIF-1α). Together, these effects activate β-catenin/HIF-1α-related pathways and enhance glycolysis, thereby promoting tumor cell proliferation, migration, and tolerance to oxaliplatin^[52]^. Exos derived from CAFs can also regulate Wnt/β-catenin signaling through distinct molecular mechanisms. On the one hand, they activate Wnt/β-catenin signaling by delivering lncRNA H19, which acts as a ceRNA to sponge miR-141. On the other hand, they enhance Wnt/β-catenin signaling by delivering miR-92a-3p, which targets and suppresses F-box and WD repeat domain-containing 7 (FBXW7). In addition, this miRNA inhibits the mitochondrial apoptotic pathway by targeting modulator of apoptosis 1 (MOAP1). These studies suggest that CAF-derived Exos can promote β-catenin-related drug resistance at multiple molecular levels, thereby enhancing tumor stemness, epithelial-mesenchymal transition, and chemoresistance^[50,51]^. In addition, Exos can activate bypass signaling by transferring transmembrane proteins. For example, Exos that carry wtEGFR and are released by tumor cells can be taken up by EGFR-mutant cells and still activate the PI3K/Akt and MAPK/ERK pathways in the presence of osimertinib, thereby enhancing tolerance to targeted therapy^[62]^. Meanwhile, membrane proteins enriched in Exos, such as integrins, can participate in interactions between Exos and recipient cells, thereby influencing cell migration and microenvironmental remodeling^[89,90]^. Overall, Exos can regulate classical signaling pathways not only at the post-transcriptional level through nucleic acid cargoes, but also through the direct transfer of membrane proteins. In doing so, they influence upstream signaling inputs and pathway activity, further contributing to the regulation of tumor progression and therapeutic responses.

#### Exosome-mediated epigenetic reprogramming

Exos can serve as important carriers of epigenetic information and mediate sustained phenotypic reprogramming in recipient cells. On the one hand, donor cells can selectively load chemically modified ncRNAs, such as m6A-modified lncRNAs, into Exos and transfer them to recipient cells. These m6A-modified lncRNAs exhibit enhanced stability and regulatory effects in recipient cells, thereby suppressing apoptosis-related processes and promoting the maintenance of the drug-resistant phenotype. For example, in multiple myeloma (MM), myeloma-associated adipocytes under tumor-induced conditions, can enhance the m6A modification of lncRNAs and package them into Exos. After transfer to MM cells, these m6A-lncRNAs suppress apoptosis and enhance tolerance to bortezomib^[59]^. On the other hand, Exos can also mediate horizontal transfer of proteins involved in RNA editing or modification, thereby altering post-transcriptional modification states in recipient cells and mediating downstream molecular network remodeling. For example, in CRC, CRC cells can transfer ADAR1 protein to tumor-associated macrophages via Exos. Within macrophages, exogenous ADAR1 mediates A-to-I editing of GLI1 mRNA, thereby enhancing GLI1-associated transcriptional activity and inducing SPP1 secretion. SPP1 then feeds back in a paracrine manner to activate the NF-κB pathway in cancer cells, ultimately enhancing their tolerance to oxaliplatin^[66]^. Overall, Exos transport not only RNA modification-related molecules, such as m6A-lncRNAs, but also RNA editing regulators, such as ADAR1. Through these mechanisms, they alter RNA stability, translation, or sequence information and establish intercellular feedforward and feedback circuits, thereby mediating sustained phenotypic reprogramming in recipient cells.

#### Exosome-mediated metabolic reprogramming

Exos can influence the metabolic networks of recipient cells through the intercellular transfer of metabolism-regulating molecules and thereby participate in tumor metabolic reprogramming. With respect to glycolysis, Exos can serve as carriers of glycolytic phenotype transmission within the tumor microenvironment. For example, in gastric cancer (GC), M2 tumor-associated macrophages (M2-TAMs) transfer lncRNA MALAT1 to cancer cells via Exos, thereby coordinately activating β-catenin and hypoxia-inducible factor 1-alpha (HIF-1α) signaling, enhancing aerobic glycolysis, and promoting oxaliplatin tolerance^[52]^. Similarly, in cisplatin-resistant NSCLC, Exos can deliver the key glycolytic enzyme pyruvate kinase M2 (PKM2) to drug-sensitive cells, thereby promoting the activation of the glycolytic program and increasing cisplatin tolerance^[49]^. In addition to promoting glycolysis, Exos can also drive a metabolic shift toward oxidative phosphorylation (OXPHOS) in tumor cells. In CRC, circ_0001610 transferred by Exos derived from drug-resistant cells relieves miR-30e-5p-mediated suppression of peroxisome proliferator-activated receptor gamma coactivator 1-alpha (PGC-1α), a master regulator of mitochondrial biogenesis, thereby enhancing mitochondrial function and OXPHOS. This metabolic reprogramming enhances stemness in CRC cells and increases their tolerance to oxaliplatin^[60]^.

## POTENTIAL CLINICAL APPLICATIONS OF EXOS IN TUMOR MULTIDRUG RESISTANCE

The roles of Exos in the formation and maintenance of the MDR suggest two areas of potential clinical application. On the one hand, their characteristic cargoes can serve as liquid biopsy biomarkers for drug resistance monitoring and dynamic assessment. On the other hand, Exos can be engineered as natural drug delivery vehicles to improve drug sensitivity or reverse drug resistance^[91,92]^.

### Exos as potential biomarkers of tumor multidrug resistance

Liquid biopsy is a non-invasive detection strategy aimed at obtaining information on tumor development and therapeutic responses by analyzing tumor-associated components in body fluids, including circulating tumor DNA (ctDNA), circulating tumor cells (CTCs), and Exos^[93]^. Compared with ctDNA and CTCs, Exos offer certain advantages in liquid biopsy. Their lipid bilayer structure helps protect cargo stability, and they are present in multiple body fluids, making repeated sampling feasible. In addition, Exos are actively secreted by living cells, and their molecular cargoes can, to some extent, reflect the biological state of donor cells, indicating their potential for dynamic monitoring of tumor evolution and therapeutic responses^[94,95]^. Based on these characteristics, circulating Exos may be used for dynamic monitoring of resistance-related information associated with certain conventional chemotherapies and targeted therapies. In addition, the membrane antigens, immunoregulatory proteins, and resistance-related ncRNAs they carry may provide a potential basis for predicting responses to monoclonal antibody therapy and monitoring therapeutic efficacy^[67,96-100]^. For example, exosomal HER2 derived from serum or plasma has been reported to be useful for assessing HER2 status, predicting benefit from trastuzumab therapy, and monitoring HER2-related treatment responses. In certain HER2-directed treatment settings, this strategy may also provide a potential basis for monitoring the efficacy of ADCs, although these findings still require further validation and refinement in future studies^[23,99,101]^.

#### Detection and analysis of exosomal cargo in liquid biopsy

**Figure 2:**
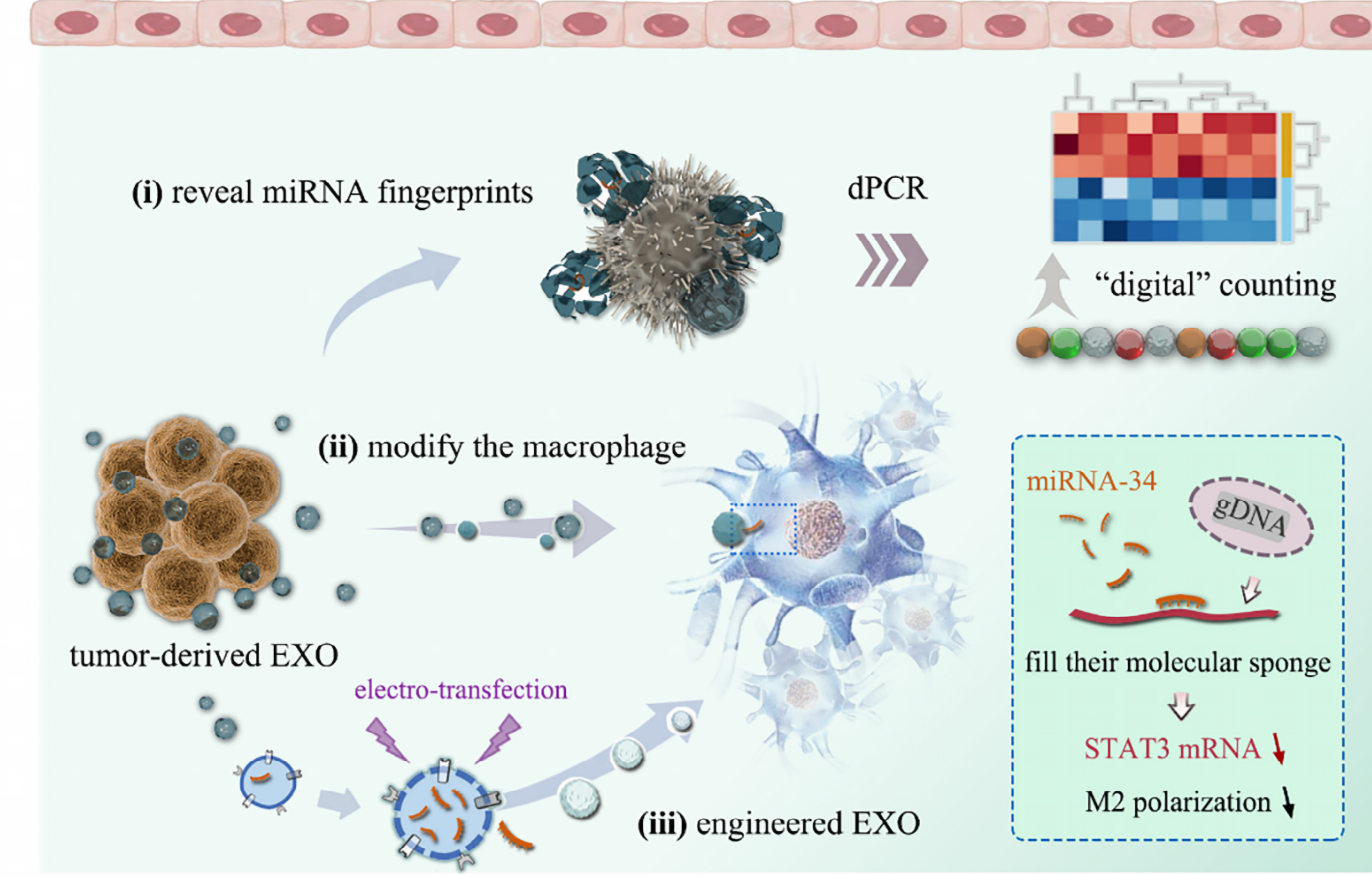
Exosome-based detection of low-abundance miRNAs with potential application in liquid biopsy and biomarker monitoring. This platform enables highly sensitive detection of low-abundance exosomal miRNAs and illustrates the potential application of exosome-based miRNA profiling in liquid biopsy and biomarker development. Adapted from Zhang *et al.*^[105]^, used under the CC BY 4.0 license. miRNAs: MicroRNAs; dPCR: digital polymerase chain reaction; EXO: exosome; gDNA: genomic DNA; STAT3: signal transducer and activator of transcription 3; mRNA: messenger RNA.

The exosomal membrane can retain characteristic antigens derived from parental cells, providing a basis for enriching tumor-related Exos from complex body fluids using immunoaffinity-based strategies. Using mass spectrometry analysis, Melo *et al.* found that glypican-1 (GPC1) was enriched on the surface of pancreatic cancer-associated Exos. In their study cohort, GPC1-positive [GPC1(+)] circulating Exos differentiated patients with pancreatic cancer from healthy individuals and those with benign pancreatic disease, suggesting their potential as candidate surface biomarkers for liquid biopsy in pancreatic cancer^[102]^. Chen *et al.* found that melanoma-derived Exos carry functional PD-L1 on their surface, enabling direct antibody-based capture and quantification of functional immunosuppressive molecules in circulation. Compared with free PD-L1 in plasma, exosomal PD-L1 exists in a membrane-bound form and is dynamically associated with responses to anti-PD-1 therapy, indicating its potential as a functional candidate biomarker for evaluating tumor immune evasion status^[83]^. In addition, Exos can provide an additional source for detecting low-abundance resistance-related nucleic acids in circulation, thereby facilitating the liquid biopsy-based detection of resistance-associated clones. For example, in NSCLC, Castellanos-Rizaldos *et al.* combined exosomal RNA/DNA with cell-free DNA (cfDNA) analysis to detect EGFR T790M mutations in plasma. This approach enhanced detection sensitivity in low-abundance samples and facilitated the detection of rare resistant clones^[103]^. Similarly, in renal cell carcinoma (RCC), Qu *et al.* found that drug-resistant cells can selectively sort and transfer lncARSR via Exos. By competitively binding miR-34 and miR-449, lncARSR upregulates AXL and c-MET expression, thereby promoting the acquisition of a drug-resistant phenotype in sensitive cells. This finding suggests that exosomal lncARSR may serve as a candidate liquid biopsy biomarker of drug resistance^[104]^. Although Exos hold promise for liquid biopsy, the low abundance of nucleic acid biomarkers in body fluids remains a challenge for highly sensitive detection. To address this bottleneck, Zhang *et al.* developed a near-infrared-responsive digital PCR strategy for highly sensitive absolute quantification of exosomal miRNAs. This platform was used to detect biomarkers such as miR-210, miR-126, and miR-30c, showing higher sensitivity than conventional methods and providing a potential technical basis for the precise detection of low-abundance exosomal nucleic acid biomarkers in liquid biopsy and biomarker monitoring^[105]^ [Figure 2].

#### Exos in drug resistance monitoring and prognostic evaluation

One of the major advantages of Exos as biomarkers is their dynamic and functional nature. Unlike ctDNA, which provides primarily genotype-level sequence information, Exos are actively secreted by viable tumor cells and cells within the tumor microenvironment, and the proteins, RNAs, lipids, and metabolism-related molecules they carry can vary with tumor origin, disease stage, and treatment response. Therefore, Exos can reflect not only certain genotypic features of tumors, but also phenotypic and non-genetic resistance-related information that ctDNA is less able to reveal, such as metabolic pathway reprogramming and functional cargo-mediated phenotypic remodeling^[94,106]^. Based on these features, Exos not only have potential as resistance-related biomarkers, but may also provide clues for elucidating the mechanisms underlying drug resistance. For example, in NSCLC models, exosomal transfer of wtEGFR can promote tolerance to osimertinib in EGFR-mutant cells, whereas hypoxia-induced exosomal delivery of pyruvate kinase M2 (PKM2) can contribute to the formation and maintenance of cisplatin resistance by promoting glycolytic reprogramming^[49,62]^. In addition, changes in the exosomal levels of these molecules further suggest their potential as candidate biomarkers for resistance detection. Therefore, exosome-based detection may not only provide early warning signals of resistance-related changes, but also offer clues for subsequent mechanism-guided therapeutic adjustment, for example, by prompting further evaluation of bypass activation or metabolism-related reprogramming and informing the exploration of corresponding combination-targeted or metabolic intervention strategies^[49,62,92,107]^. Moreover, exosomal molecular profiles may serve as candidate indicators for dynamic monitoring of resistance evolution. For example, in small cell lung cancer (SCLC), plasma exosomal miR-92b-3p levels are elevated in patients with chemoresistant disease and decrease after remission, suggesting their potential utility in tracking the evolution of chemoresistance^[108]^. A similar phenomenon has also been reported in the monitoring of chemoresistance in lung adenocarcinoma. Studies have shown that chemoresistance-related long non-coding RNA (lncCRLA) is highly expressed in lung adenocarcinoma cells with a mesenchymal phenotype and can be released into the circulation via Exos. Plasma exosomal lncCRLA levels show high concordance with expression in tumor tissues, suggesting that it may be useful not only for evaluating chemotherapy response, but also for dynamically reflecting disease progression and recurrence-related risk^[109]^. Notably, the value of Exos as dynamic liquid biopsy biomarkers is not limited to chemotherapy and targeted therapy. In patients receiving monoclonal antibody treatment, circulating Exos carrying target antigens, immunoregulatory proteins, and related RNA molecules have also shown potential as candidate liquid biopsy markers for monitoring treatment response and resistance-associated changes. In certain HER2-related therapeutic settings, this strategy may likewise provide clues for assessing the efficacy of ADCs^[98,99]^.

### Exosome-related strategies for reversing drug resistance

#### Inhibition of exosome biogenesis and secretion

One direct strategy for MDR reversal is to block exosome-mediated intercellular communication by inhibiting exosome biogenesis or release. GW4869 is a commonly used inhibitor of exosome release in experimental studies. By suppressing the neutral sphingomyelinase-related pathway, it interferes with ceramide-dependent exosome biogenesis, thereby reducing exosome-mediated intercellular molecular transfer and signal transmission^[110,111]^. In addition, the natural product psoralen has been reported to inhibit exosome biogenesis and secretion in drug-resistant breast cancer cells (MCF-7/ADR), thereby reducing the horizontal transfer of resistance traits to sensitive cells and suggesting its potential for MDR reversal^[112]^. In the context of drug repositioning, sulfisoxazole was initially identified through screening as an endothelin receptor A (ETA)-related inhibitory molecule, and has been reported to suppress the release of small extracellular vesicles from breast cancer cells^[113]^. However, whether it can stably inhibit the secretion of Exos or small extracellular vesicles remains controversial, as subsequent studies have yielded inconsistent results. In other studies, this agent has been shown to reduce PD-L1 levels on circulating Exos, thereby, to some extent, enhancing the response to anti-PD-1 therapy and boosting antitumor immune responses^[114]^.

#### Blocking the selective sorting and loading of resistance-related cargo

Although inhibiting exosome secretion can reduce the spread of pathological signals, broadly blocking exosome release may also disrupt normal intercellular communication. Therefore, a more refined strategy is to selectively interfere with the loading of resistance-related cargo rather than completely suppress exosome biogenesis. This strategy can be achieved by targeting key sorting nodes. For example, the RNA-binding protein YBX1 is an important regulator of exosomal RNA sorting and recognizes and promotes the enrichment of certain miRNAs, such as miR-223, into Exos. Thus, targeting YBX1 function may help reduce the loading efficiency of some resistance-related nucleic acid cargoes^[72]^. In addition, m6A modification is considered to be involved in cargo sorting. A study by Wang *et al.* showed that specific methyltransferases, such as METTL7A, can increase the m6A modification level of resistance-related lncRNAs, thereby promoting their loading into Exos and transfer to recipient cells, ultimately facilitating the development of a drug-resistant phenotype^[59]^. Meanwhile, m6A-related reader proteins and RNA-binding proteins, such as hnRNPA2B1, also play important roles in the recognition of modified RNAs and their sorting into Exos^[73,74]^. Therefore, targeting methyltransferase activity or interfering with the function of relevant reader proteins may represent a potential strategy for reducing the loading of resistance-related exosomal cargo. It should also be noted that, in addition to directly interfering with cargo sorting, blocking membrane fusion and release steps closely linked to post-sorting export may also reduce the final output of resistance-related cargo. Lauwers *et al.* demonstrated that Hsp90 has a key membrane-remodeling function and promotes the fusion of multivesicular bodies with the plasma membrane, thereby enhancing exosome release; thus, targeting Hsp90 may serve as an auxiliary strategy to suppress the export of resistance-related cargo^[115]^.

#### Engineered exosome-based strategies for reversing drug resistance

**Figure 3:**
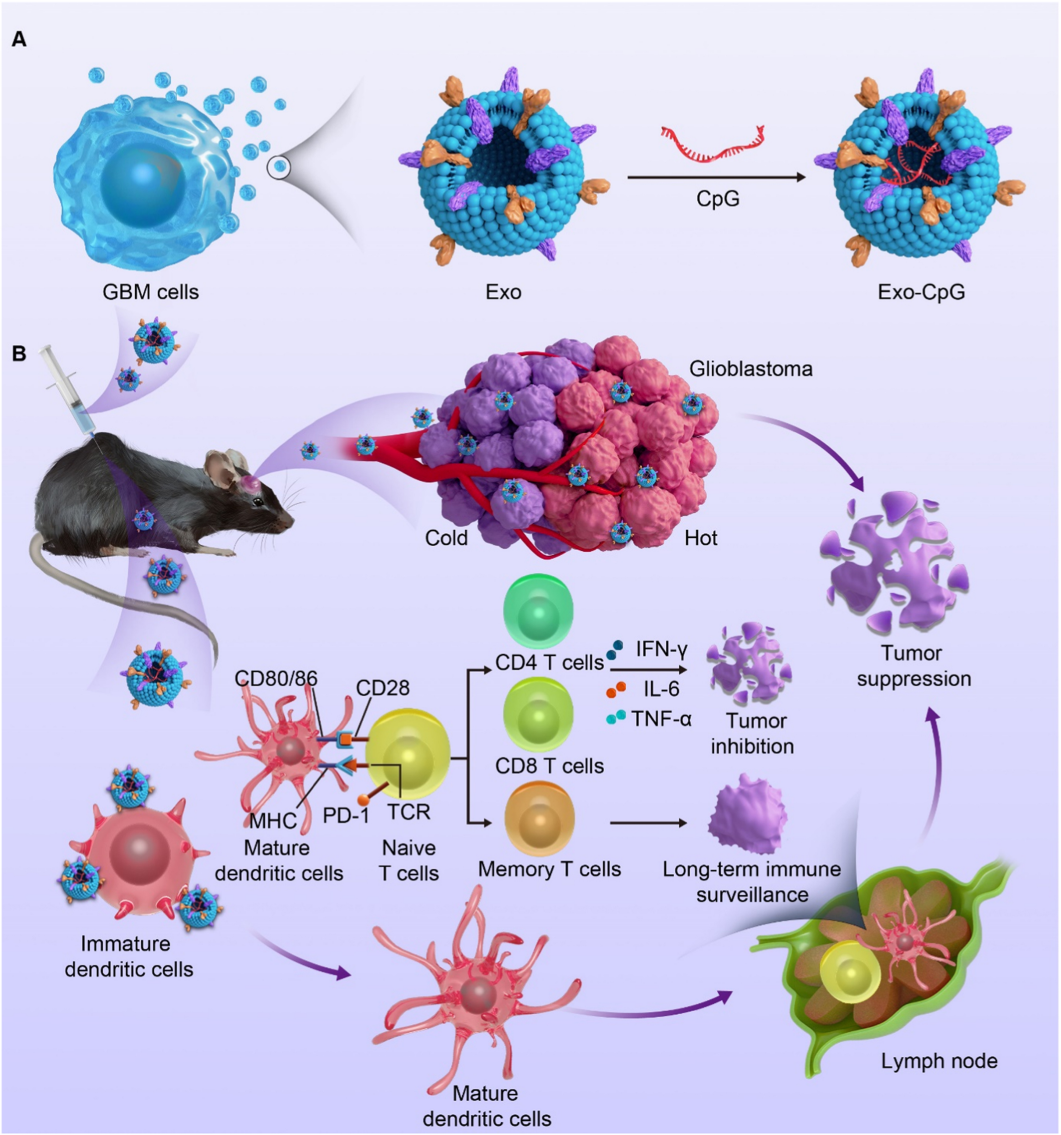
Schematic illustration of the tumor cell-derived engineered exosome Exo-CpG for enhancing immunotherapy against glioblastoma. (A) Exos were isolated from glioblastoma (GBM) cells and loaded with the CpG adjuvant to construct the engineered exosome Exo-CpG; (B) After entering brain tumor tissue, Exo-CpG enhances antigen presentation and promotes dendritic cell (DC) activation, thereby inducing CD4+ and CD8+ T-cell responses and related cytokine secretion. These effects suppress orthotopic tumor growth and may also enhance immune responses against recurrent tumors. Adapted from Li *et al.*^[126]^, used under the CC BY 4.0 license. Exo: Exosome; MHC: major histocompatibility complex; PD-1: programmed cell death protein 1; TCR: T cell receptor; IFN-γ: interferon-gamma; IL-6: interleukin-6; TNF-α: tumor necrosis factor-alpha.

**Figure 4:**
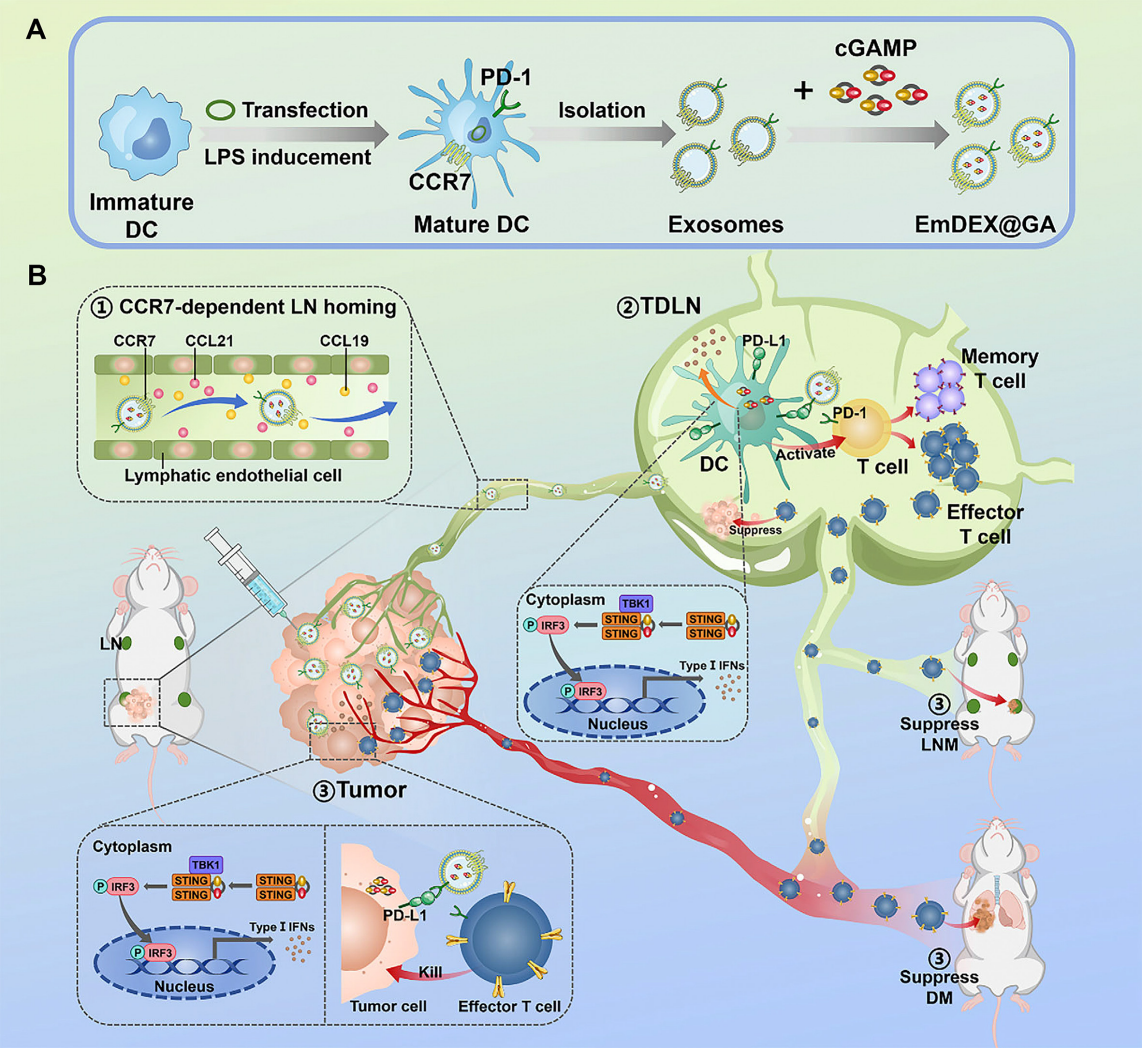
Schematic illustration of how the engineered dendritic cell-derived exosome EmDEX@GA modulates the lymph node immune microenvironment. (A) Dendritic cells (DCs) were genetically engineered to overexpress CCR7 and PD-1. The engineered Exos secreted by these cells were then collected and loaded with the STING agonist Gauron to construct EmDEX@GA; (B) Through surface CCR7, EmDEX@GA accumulates in tumor-draining lymph nodes (TDLNs) and, via local PD-1/PD-L1 blockade and STING pathway activation, modulates the lymph node immune microenvironment, thereby enhancing antitumor immune responses and suppressing tumor metastasis. Adapted from Wang *et al.*^[127]^, used under the CC BY 4.0 license. CCR7: C-C chemokine receptor type 7; PD-1: programmed cell death protein 1; Exos: exosomes; STING: stimulator of interferon genes; PD-L1: programmed death-ligand 1; cGAMP: cyclic GMP-AMP; GMP: guanosine monophosphate; AMP: adenosine monophosphate; CCL: C-C motif chemokine ligand; LN: lymph node; TBK1: TANK-binding kinase 1; IRF3: interferon regulatory factor 3; IFNs: interferons; LNM: lymph node metastasis; DM: distant metastasis.

Engineered Exos constitute a promising strategy for reversing MDR by serving as nanoplatforms for therapeutic cargo delivery. This approach exploits Exos as natural drug delivery systems (DDSs), in which chemotherapeutic agents^[116]^, functional nucleic acids^[91]^, or immunostimulatory agents^[117]^ can be loaded into Exos through genetic engineering of parental cells or physicochemical loading techniques, thereby enhancing their potential for targeted delivery to and intervention in drug-resistant tumors. Compared with conventional synthetic liposomes, engineered Exos offer certain inherent delivery advantages. First, they exhibit favorable biocompatibility^[118]^. Second, they can, to some extent, retain membrane characteristics derived from their parental cells, which may help improve circulatory stability in vivo and the efficiency of targeted delivery^[116,119]^. These intrinsic membrane features contribute to more efficient drug delivery to drug-resistant tumors and may, to some extent, improve therapeutic responses. Exos, as natural gene delivery vehicles, encapsulate and deliver siRNAs or anti-miRNAs, thereby intervening in drug resistance-related gene networks at the post-transcriptional level^[91,120]^. To further improve delivery efficiency and tumor selectivity, active targeting modification is considered an important strategy. For example, Exos modified with the iRGD peptide for delivery of CPT1A siRNA promote reversal of the oxaliplatin-resistant phenotype in a colon cancer model^[120]^. Likewise, engineered Exos delivering siBRIX1 can induce nucleolar stress and inhibit colorectal cancer growth, while also enhancing the therapeutic efficacy of 5-FU^[121]^. In addition, Exos derived from specific cell sources may further improve delivery compatibility and enhance the efficacy of resistance-targeted interventions. For example, BM-MSC-derived Exos delivering siGRP78 can suppress the endoplasmic reticulum chaperone GRP78 at the post-transcriptional level, thereby enhancing the sensitivity of hepatocellular carcinoma to sorafenib^[122]^. Owing to their unique vesicular structure, Exos also serve as a promising platform for the co-delivery of nucleic acids and small-molecule drugs, with the potential to achieve synergistic effects through complementary mechanisms^[123]^. For example, engineered Exos modified with a HER2 affibody and co-loaded with 5-FU and a miR-21 inhibitor have been shown to restore PTEN and hMSH2 expression while suppressing oncogenic miRNA activity, thereby promoting the re-sensitization of colorectal cancer to 5-FU^[91]^. In addition, engineered Exos show potential value in overcoming therapeutic barriers in drug-resistant tumors. For instance, engineered Exos derived from a drug-resistant T-ALL cell line and loaded with isoliquiritigenin can exploit their bone marrow tropism to induce tumor cell apoptosis, autophagy, and DNA damage, thereby helping overcome drug-resistant T-ALL in a patient-derived xenograft (PDX) model^[124]^. Beyond the direct delivery of sensitizing molecules or cargoes that reverse drug resistance, engineered Exos can also indirectly improve therapeutic responses in drug-resistant tumors by remodeling antitumor immune responses. They also serve as an important delivery platform for enhancing responses to immunotherapy. For example, engineered dendritic cell-derived Exos co-loaded with CML tumor antigens and RAE-1γ can synergistically activate NK cells and T cells through the NKG2D/NKG2D-L pathway, thereby enhancing the clearance of CML cells carrying the T315I mutation^[125]^. In addition, GBM-derived engineered Exos loaded with the CpG adjuvant have been shown to enhance immunotherapeutic efficacy and suppress tumor growth in orthotopic brain tumor models and recurrent tumor models^[126]^ [Figure 3]. On the other hand, remodeling the local immune microenvironment is also an important strategy for improving therapeutic responses and overcoming immunosuppression. Wang *et al.* developed an engineered dendritic cell-derived exosome platform, EmDEX@GA, which achieves efficient accumulation in tumor-draining lymph nodes (TDLNs) through the active homing effect of the surface chemokine receptor CCR7. This system enables local immune checkpoint intervention by displaying PD-1 on the exosome surface, while simultaneously activating innate immune signaling pathways through the loaded STING agonist, thereby reshaping the immunosuppressive microenvironment in TDLNs and enhancing antitumor immune responses^[127]^ [Figure 4].

#### Inhibition of exosome uptake and targeted clearance

Blocking exosome-mediated signal transmission can be approached at two levels: extracellular neutralization and inhibition of uptake by recipient cells. On the one hand, specific antibodies, such as anti-PD-L1 antibodies, are used to bind circulating Exos carrying PD-L1, thereby blocking their interaction with PD-1 receptors on the surface of T cells, attenuating exosome-mediated systemic immunosuppression, and potentially restoring the antitumor function of T cells to some extent^[83]^. On the other hand, inhibiting the uptake of Exos by recipient cells also represents a potential strategy for interrupting the transmission of drug resistance. Studies have shown that small-molecule inhibitors, such as Dynasore, which suppresses dynamin-dependent endocytosis, reduce cellular uptake of Exos to some extent, thereby weakening exosome-mediated transfer of resistance-related signals and, to a certain extent, enhancing tumor sensitivity to chemotherapeutic agents^[128]^. Given the central role of Exos in the dissemination of drug resistance and remodeling of the tumor microenvironment, current intervention strategies for reversing MDR mainly include inhibiting exosome biogenesis and secretion, blocking the selective sorting and loading of resistance-related cargo, using engineered Exos to deliver resistance-reversing agents, and inhibiting exosome uptake or promoting their targeted clearance, as summarized in Table 3.

**Table 3:** Exosome-targeted therapeutic strategies for reversing tumor multidrug resistance

**Strategy category**	**Specific approach/drug**	**Mechanism of action**	**Target cancer**	**Resistance type**	**References**
Inhibition of exosome biogenesis and secretion	Psoralen	Inhibits exosome formation and secretion from drug-resistant cells, thereby blocking the spread of resistant phenotypes	Breast cancer	MDR	[112]
	SFX	Targets ETA to inhibit the secretion of sEVs/Exos	Breast cancer	Exosome-mediated therapeutic resistance	[113]
	Sulfisoxazole combined with anti-PD-1 therapy	Reduces exosomal PD-L1 and alleviates T-cell exhaustion	Breast cancer, colon cancer	Anti-PD-1 immunotherapy resistance	[114]
Blocking the selective sorting and loading of resistance-associated cargoes	Targeting the METTL7A/m6A modification-associated process	Reduces the exosomal loading of resistance-associated lncRNAs	Multiple myeloma	Bortezomib/melphalan/carfilzomib resistance	[59]
Engineered exosome-based delivery of resistance-reversing agents	iRGD-modified Exos delivering CPT1A siRNA	Silences CPT1A, inhibits FAO, and restores drug sensitivity	Colon cancer	Oxaliplatin resistance	[120]
	Engineered Exos delivering siBRIX1	Silences BRIX1, induces nucleolar stress, and enhances the chemotherapeutic response	CRC	5-FU resistance	[121]
	BM-MSC-derived Exos delivering siGRP78 combined with sorafenib	Silences GRP78 and restores sorafenib sensitivity	HCC	Sorafenib resistance	[122]
	Engineered Exos co-delivering 5-FU and a miR-21 inhibitor	Downregulates miR-21, restores PTEN/hMSH2-associated signaling, and enhances the response to 5-FU	Colon cancer	5-FU resistance	[91]
	Engineered Exos loaded with isoliquiritigenin	Induces apoptosis, autophagy, and DNA damage through bone marrow-targeted delivery	T-ALL	Drug-resistant T-ALL	[124]
	Modified DC-derived Exos loaded with CML tumor antigens and RAE-1γ	Activates NK cells and T cells via the NKG2D/NKG2D-L pathway	CML	T315I mutation-associated TKI resistance	[125]
	Exo-CpG	Enhances local antitumor immunity and improves therapeutic responses in recurrent tumors	Glioblastoma	Immunotherapy tolerance/recurrent disease-associated therapeutic resistance	[126]
	EmDEX@GA	Targets TDLNs while simultaneously enabling PD-1/PD-L1 blockade and STING activation, thereby remodeling the immunosuppressive microenvironment	Breast cancer	Immunosuppression-associated therapeutic tolerance/insufficient immunotherapeutic response associated with metastasis	[127]
Blocking exosome uptake and targeted clearance	Dynasore combined with bortezomib	Inhibits dynamin-dependent endocytosis, reduces the uptake of stromal cell-derived EVs by myeloma cells, and restores drug sensitivity	Multiple myeloma	Bortezomib resistance	[128]

Exos: Exosomes; MDR: multidrug resistance; SFX: sulfisoxazole; ETA: endothelin receptor A; sEVs: small extracellular vesicles; PD-L1: programmed death-ligand 1; PD-1: programmed cell death protein 1; lncRNA: long non-coding RNA; BM-MSC: bone marrow mesenchymal stem cell; HCC: hepatocellular carcinoma; 5-FU: 5-fluorouracil; T-ALL: T-cell acute lymphoblastic leukemia; DC: dendritic cell; CML: chronic myeloid leukemia; NK: natural killer; NKG2D: natural killer group 2D; NKG2D-L: NKG2D ligand; TDLNs: tumor-draining lymph nodes; STING: stimulator of interferon genes; EVs: extracellular vesicles; CRC: colorectal cancer; TKI: tyrosine kinase inhibitor; FAO: fatty acid oxidation.

## RESEARCH CHALLENGES AND FUTURE PERSPECTIVES

Although studies on exosome-mediated MDR have provided new insights into tumor drug resistance, multiple challenges remain in translating these findings from basic research into clinical applications^[129,130]^. Future research may need to move beyond static descriptions of single molecular mechanisms toward dynamic analyses of vesicle communication networks and their spatiotemporal heterogeneity, while integrating systematic intervention strategies, thereby achieving a more comprehensive understanding of exosome-mediated resistance networks and exploring potential therapeutic avenues^[131]^. One of the central barriers to clinical translation lies in the lack of standardized manufacturing processes and high-precision detection methods. First, major challenges remain in clinical-grade production: existing isolation techniques, such as ultracentrifugation and polymer-based precipitation, cannot simultaneously ensure purity, yield, and scalability, and there is still no unified and widely accepted GMP (Good Manufacturing Practice)-grade quality control standard. As a result, the comparability and reproducibility of data across different studies remain limited^[130,132,133]^. Second, the “signal interference” caused by heterogeneity remains a major challenge. Exos in liquid biopsy samples are highly heterogeneous in origin and represent a mixture of secretions from multiple cell populations within the TME^[44,92]^. Exos from different sources may carry cargoes with markedly different, or even opposing, functions. For example, CAF-derived Exos may carry resistance-promoting molecules, whereas BMSC-derived miR-193a may enhance drug sensitivity and promote reversal of the resistant phenotype^[68,134]^. Traditional bulk analysis often masks critical information from key pathogenic subpopulations by averaging the overall signal. Therefore, the development of high-throughput single-vesicle analysis technologies capable of tracing vesicle origin and profiling cargo at the individual particle level represents an important direction for improving diagnostic specificity^[135]^. In addition, the mechanisms by which Exos mediate drug resistance are not universal, but instead exhibit marked tissue and microenvironment specificity. For example, CAF-derived Exos in colorectal cancer can promote drug resistance through Wnt/β-catenin-related pathways^[50,51]^, whereas in breast cancer, miR-22 carried by CAF-derived Exos can induce resistance to endocrine therapy by suppressing ERα^[136]^. Therefore, elucidating this context dependence and the underlying tissue-specific regulatory mechanisms is an important foundation for advancing precision intervention. Third, resistance transmission is dynamically evolving. Exosome-mediated dissemination of drug resistance may change in response to drug pressure and shifts in cellular state, and observations at a single time point may not fully capture the actual process^[62]^. Along the temporal dimension, chemotherapy or radiotherapy itself can trigger changes in exosome release and cargo composition. For example, chemotherapy can induce breast cancer cells to release Exos enriched in miR-378a-3p, whereas radiotherapy can induce GBM cells to release vesicular secretions carrying miR-603, thereby promoting therapeutic resistance^[137,138]^. These findings suggest that the transmission of resistance-related signals may be further amplified during the post-treatment stress phase. Along the spatial dimension, the heterogeneous microenvironment created by intratumoral oxygen and nutrient gradients may influence exosome secretion and cargo composition through hypoxia/HIF-related pathways, thereby promoting the intratumoral spread of resistance-promoting signals^[139]^.

Given the complexity of MDR, a single biomarker or monotherapy is often insufficient to address this challenge, and future clinical strategies may require multidimensional assessment and combined intervention^[17,140]^. On the one hand, the development of personalized exosome-based biomarker panels may be considered. Given the highly adaptive and evolving nature of MDR, a single biomarker is often unable to capture its full landscape^[17]^. Therefore, future monitoring strategies may therefore incorporate multidimensional combinations of exosomal cargo features to reflect tumor status and its potential evolutionary trajectories more comprehensively. Recent studies suggest that exosome profiling that integrates high-dimensional information, such as proteomic features, may improve the accuracy of tumor state identification and compensate for the limited specificity of single biomarkers^[141]^. Building on this, developing multidimensional feature combinations or multi-omics panels based on exosomal cargo may enable early warning and help distinguish different resistance-related biological processes, such as enhanced drug efflux or metabolic reprogramming^[142-144]^. On the other hand, given the networked nature of MDR, a combined therapeutic strategy that simultaneously “kills tumor cells” and “cuts off communication” may be considered. In combination with chemotherapy, the combined inhibition of exosome release or uptake, or blockade of CAF-related resistance pathways such as Wnt/β-catenin and H19, may weaken the resistance-supporting effects of stromal cells on tumor cells^[51,145]^. In immunotherapy, combined suppression of exosomal PD-L1 may alleviate systemic immunosuppression and enhance systemic antitumor effects^[83,84]^.

## CONCLUSION

This review systematically summarizes the major mechanisms by which Exos participate in the intercellular spread of MDR as key mediators of communication within the TME. It also discusses their translational potential in dynamic resistance monitoring and in engineered delivery strategies aimed at overcoming drug resistance. Studies on exosome-mediated intercellular communication have deepened our understanding of the development and progression of MDR, expanding resistance research from a sole focus on tumor cell-intrinsic abnormalities to the broader level of TME-driven networked interactions. As key carriers within this complex network, Exos deliver molecular cargo such as ncRNAs and functional proteins, thereby not only propagating resistant phenotypes between cells but also influencing the metabolic and immune states of the TME, ultimately promoting the establishment and maintenance of therapeutic tolerance. On this basis, future clinical strategies may integrate “tumor cell killing” with “communication blockade.” From a diagnostic perspective, developing exosome-based dynamic functional monitoring may help capture the evolution of resistance in real time. From a therapeutic perspective, one approach is to weaken resistance-supportive effects by inhibiting exosome-mediated transmission of resistance-related signals, whereas another is to develop engineered Exos as targeted delivery carriers. Overall, intervening in exosome-mediated intercellular communication networks may offer a potential stretegy for weakening adaptive drug resistance in tumors and improving the efficacy of precision therapy.

## DECLARATIONS

### Copyright

© The Author(s) 2026.
